# Preparation of porous Fe_2_O_3_ nanorods-reduced graphene oxide nanohybrids and their excellent microwave absorption properties

**DOI:** 10.1038/s41598-017-11131-1

**Published:** 2017-09-11

**Authors:** Qi Hu, Xiaosi Qi, Hongbo Cai, Ren Xie, Liu Long, Zhongchen Bai, Yang Jiang, Shuijie Qin, Wei Zhong, Youwei Du

**Affiliations:** 1 College of Physics, Guizhou University, Guiyang, 550025 People’s Republic of China; 2 Collaborative Innovation Center of Advanced Microstructures, Nanjing National Laboratory of Microstructures and Jiangsu Provincial Laboratory for NanoTechnology, Nanjing University, Nanjing, 210093 People’s Republic of China

## Abstract

In this paper, α-Fe_2_O_3_ nanoparticles (NPs)-reduced graphene oxide (RGO), α-FeOOH nanorods (NRs)-RGO and porous α-Fe_2_O_3_ NRs-RGO could be selectively synthesized by hydrothermal method. The investigations indicated that the obtained α-Fe_2_O_3_ NPs, α-FeOOH NRs and porous α-Fe_2_O_3_ NRs were either attached on the surface of RGO sheets or coated uniformly by the RGO sheets. And the as-prepared nanohybrids exhibited excellent microwave absorption performance, which was proved to be ascribed to the quarter-wavelength matching model. The optimum reflection loss (RL) values for α-Fe_2_O_3_ NPs-RGO, α-FeOOH NRs-RGO and porous α-Fe_2_O_3_ NRs-RGO were ca. −32.3, −37.4 and −71.4 dB, respectively. Moreover, compared to the obtained α-Fe_2_O_3_ NPs-RGO and α-FeOOH NRs-RGO, the as-prepared porous α-Fe_2_O_3_ NRs-RGO nanohybrids exhibited enhanced microwave absorption properties because of their special structure and synergetic effect. The possible enhanced microwave absorption mechanisms were discussed in details. Our results confirmed that the geometrical morphology had a great influence on their microwave absorption properties, which provided a promising approach to exploit high performance microwave absorbing materials.

## Introduction

In recent decades, with the rapidly extensive application of wireless equipment, radar systems and local area networks, etc, electromagnetic (EM) interference, EM radiation and EM compatibility have become the serious problems, which not only are harmful to human and the operation of electronic devices, but also influence the development of modern military^1–4^. As a kind of functional material, microwave absorbing materials (MAMs) can effectively absorb EM waves by either dissipating EM wave loss or converting EM energy into thermal energy. Hence, high performance MAMs with light weight, strong absorption ability and wide absorption frequency are highly desired. According to EM energy conversion principle, the traditional single dielectric/magnetic loss absorbers such as ferrite, ZnO and Fe_3_O_4_ are difficult to meet this condition due to the mismatch in the values of complex permittivity $({{\boldsymbol{\varepsilon }}}_{{\boldsymbol{r}}}={\boldsymbol{\varepsilon }}{\boldsymbol{^{\prime} }}-j{\boldsymbol{\varepsilon }}{\boldsymbol{^{\prime\prime} }})$ and complex permeability $({{\boldsymbol{\mu }}}_{{\boldsymbol{r}}}={\boldsymbol{\mu }}{\boldsymbol{^{\prime} }}-{\boldsymbol{j}}{\boldsymbol{\mu }}{\boldsymbol{^{\prime\prime} }})$ ^5–8^. One of the effective ways to solve the problem is to couple dielectric materials with nanostructured materials^9–11^. Therefore, various hybrids have been investigated in order to reach the targets over the past years^12–16^. Among these hybrids, carbon-based hybrids own advantages such as low density, good chemical stability and high complex permittivity value, which may improve the microwave absorption properties and EM interference shielding effect^17–19^.

Recently, graphene (G), as a new kind of carbon material, has attracted tremendous scientific attention in recent years because of its outstanding physical and chemical properties such as the excellent thermal and electronic conductivity, huge specific surface area, and so on^20–22^. Therefore, anchoring transition metal oxides onto G-based matrix will be a promising strategy to develop high performance MAMs^23^. Moreover, the previous theoretical studies indicated that the interfacial electronic interaction between metal and G could make G show some novel magnetic and electric properties^24, 25^. Therefore, different categories of G-based nanohybrids have been developed to improve microwave absorption properties in the recent years^26–29^. However, the focus of these studies is mainly on the particles. And the recently reported results indicate that the crystal structure, size and special geometrical morphology also may have an influence on their microwave absorption properties, and these related studies were seldom reported before^30, 31^.

In this paper, we develop a simple strategy to selectively synthesize heterostructured α-Fe_2_O_3_ nanoparticles (NPs)-reduced graphene oxide (RGO), α-FeOOH nanorods (NRs)-RGO and porous α-Fe_2_O_3_ NRs-RGO nanohybrids by controlling the categories of the initial reactant, respectively. Through the detailed investigations, we find that the as-synthesized heterostructured nanohybrids improve greatly their microwave absorption capabilities compared with those of the single composition of FeOOH or graphene oxide (GO). More importantly, compared to α-Fe_2_O_3_ NPs-RGO and α-FeOOH NRs-RGO, the as-synthesized porous α-Fe_2_O_3_ NRs-RGO nanohybrids exhibit enhanced microwave absorption performance.

## Results

**Figure 1:**
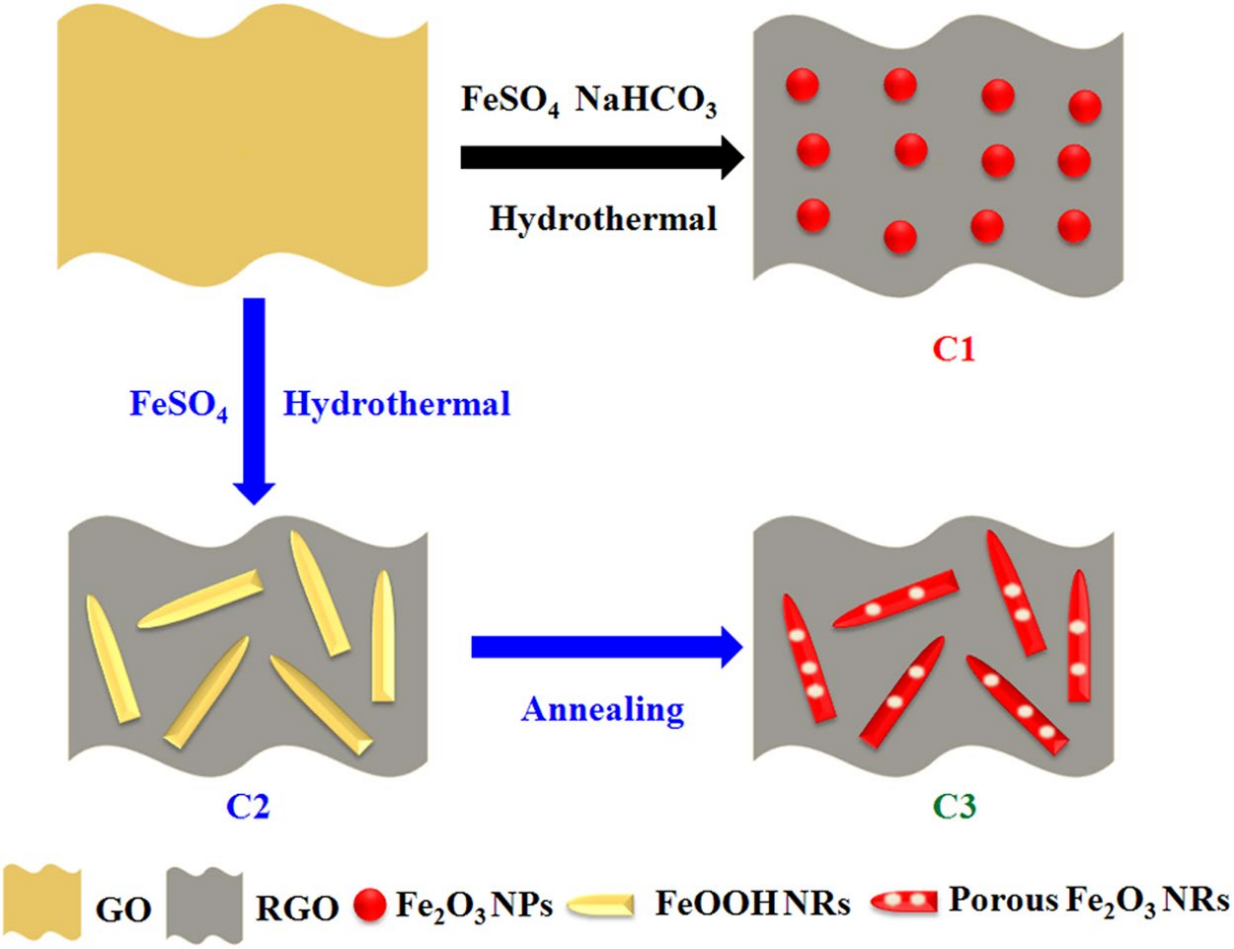
Schematic diagram for the synthesis process of Fe based-RGO nanohybrids.

**Figure 2:**
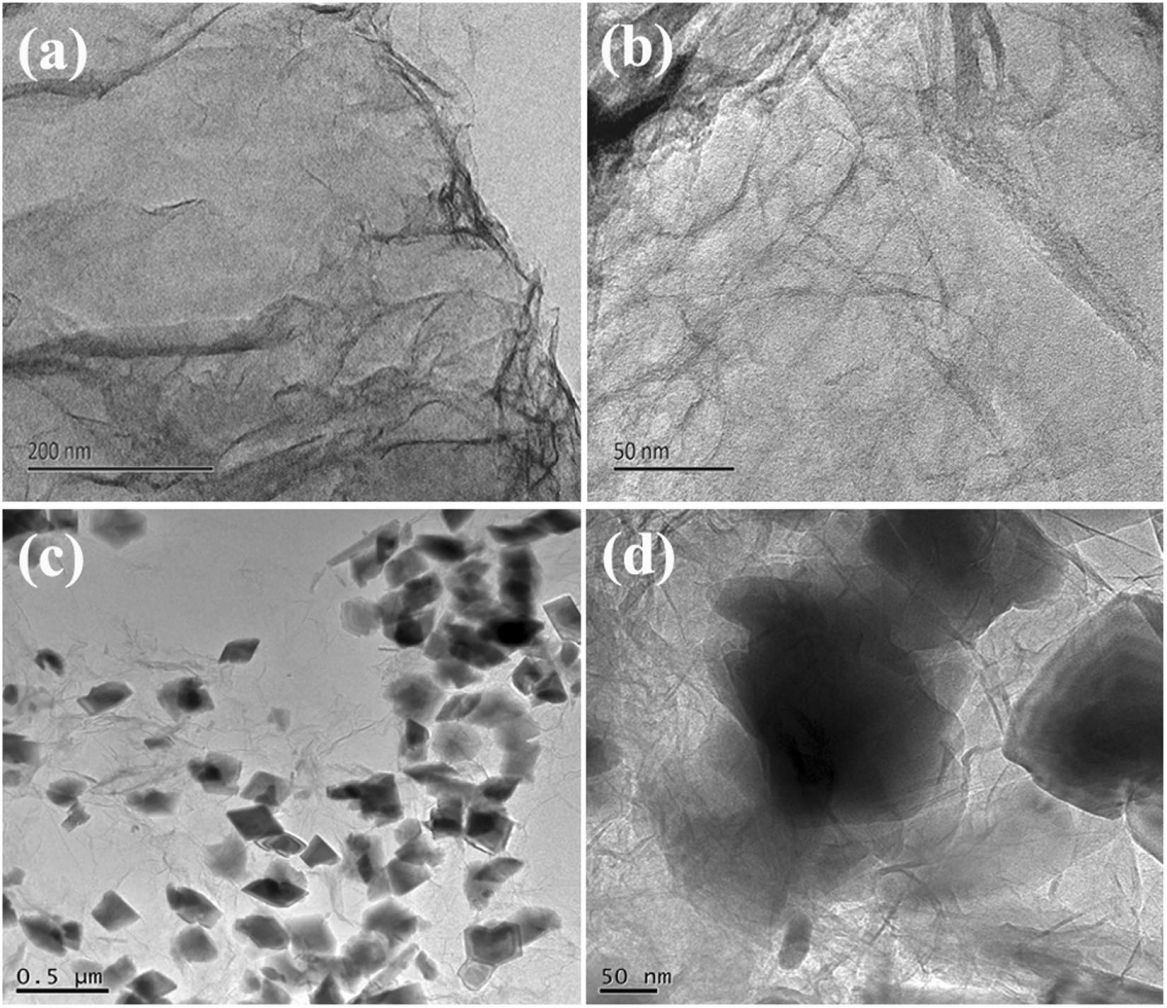
TEM images of (**a**,**b**) GO, and (**c**,**d**) C1.

The preparation process of Fe based-RGO nanohybrids is illustrated in Fig. 1. When the solutions of FeSO_4_, NaHCO_3_ and GO are mixed, the redox reaction during the hydrothermal treatment brings the formation of heterostructured Fe_2_O_3_ NPs-RGO nanohybrids (denoted as C1). However, if only the solutions of FeSO_4_ and GO are mixed, heterostructured FeOOH NRs-RGO nanohybrids (denoted as C2) can be synthesized because Fe^2+^ cations from FeSO_4_ can favourably binding with oxygen-containing on GO sheets during the hydrothermal treatment. Similar to the previously reported results^32, 33^, porous Fe_2_O_3_ NRs-RGO nanohybrids (denoted as C3) could be obtained after the annealing treatment of C2. Moreover, for comparison, FeOOH NRs are also synthesized (detailed experiment, see supporting information). Figure 2 presents the TEM images of GO and C1. As shown in Fig. 2a and b, the wrinkled and transparent paper-like structures of GO can be observed clearly, indicating that GO is a few atomic layers in thickness and good quality^34^. The morphology of GO is very similar to the previous report^35^. The TEM observation (as shown in Fig. 2c and d) indicates that the obtained C1 consists of two-dimensional RGO sheets and Fe_2_O_3_ NPs, and the as-prepared heterostructured Fe_2_O_3_-RGO nanohybrids exhibit the wrinkled paper-like structure, same to the characteristic feature of GO sheets. Moreover, the Fe_2_O_3_ NPs with size in the range of 50–200 nm are well distributed and decorated on RGO or coated by the RGO sheets. There is no apparent aggregation of Fe_2_O_3_ NPs on the RGO sheets. Compared with the recently reported work by Zhang *et al*.^36^, the proposed route here is much more simple and effective.

**Figure 3:**
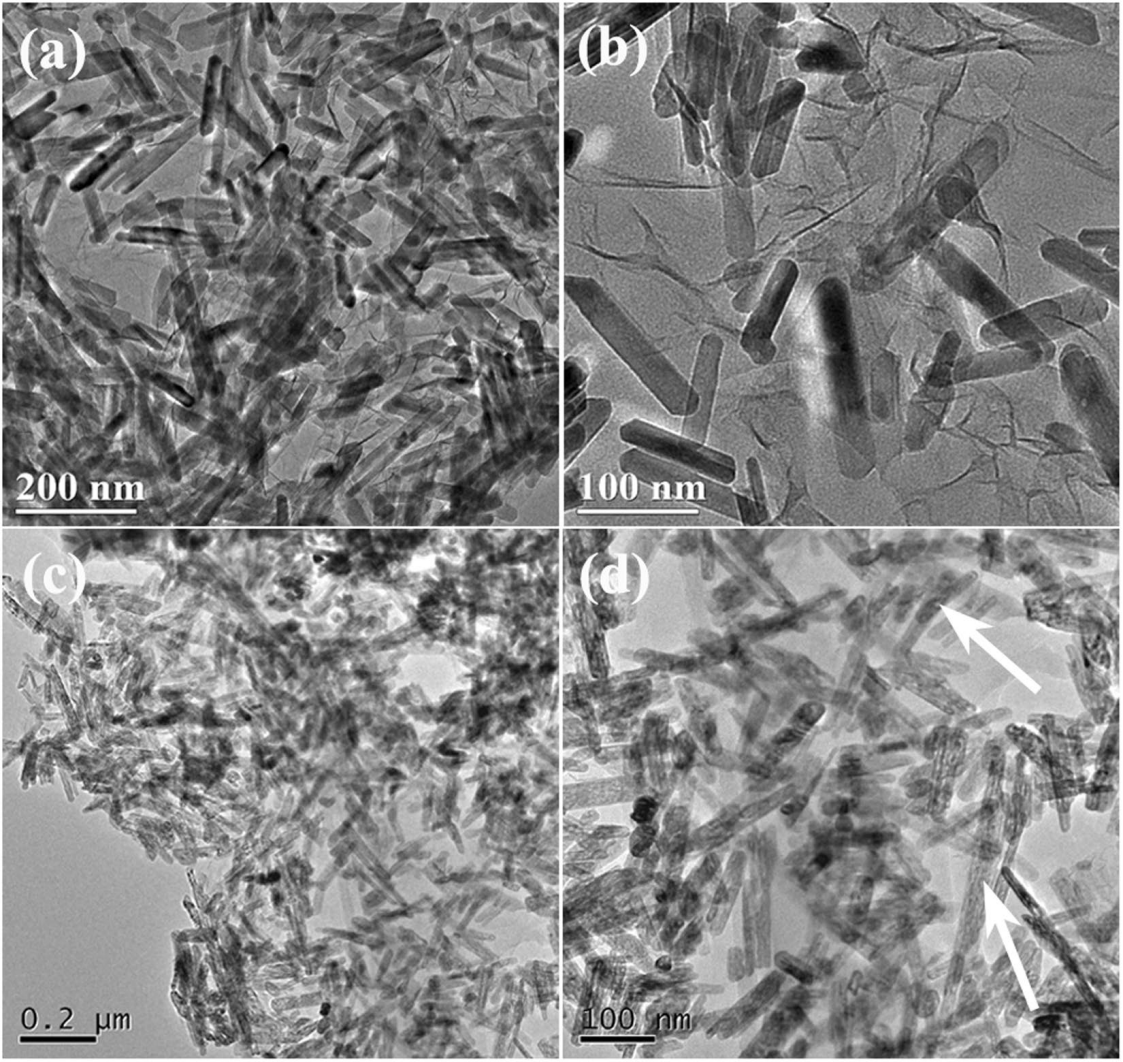
TEM images of (**a**,**b**) C2, and (**c**,**d**) C3.

Figure 3 displays the TEM images of the as-prepared C2 and C3. As shown in Fig. 3a and b, the flexible two-dimensional RGO sheets and FeOOH NRs can be observed clearly over the obtained C2. Similar to MnO_2_ NRs-RGO and MnO_2_@Fe-G reported elsewhere^37, 38^, the obtained FeOOH NRs, show very uniform sizes in diameter and length, load tightly on the thin flaky RGO sheets or are enwrapped by the RGO sheets. The TEM investigation (as shown in Fig. 3c and d) indicates that the as-synthesized C3 contains the wrinkled paper-like structure of RGO and porous Fe_2_O_3_ NRs (as indicated by the arrows in Fig. 3d). Same to those of C1 and C2, the obtained porous Fe_2_O_3_ NRs exhibit uniform sizes and are anchored on RGO surface or coated in the RGO sheets without serious aggregation. Moreover, as shown in Figure S1, the porous structure of Fe_2_O_3_ NRs can also be confirmed further by the N_2_ adsorption and desorption isotherms. Compared to that of C2, one can find that the as-prepared C3 exhibits an evidently enhanced BET surface area. Generally, compared to the previously reported iron oxides@RGO^36^, this proposed route not only can control the category of iron oxide, but also adjusts its morphology.

**Figure 4:**
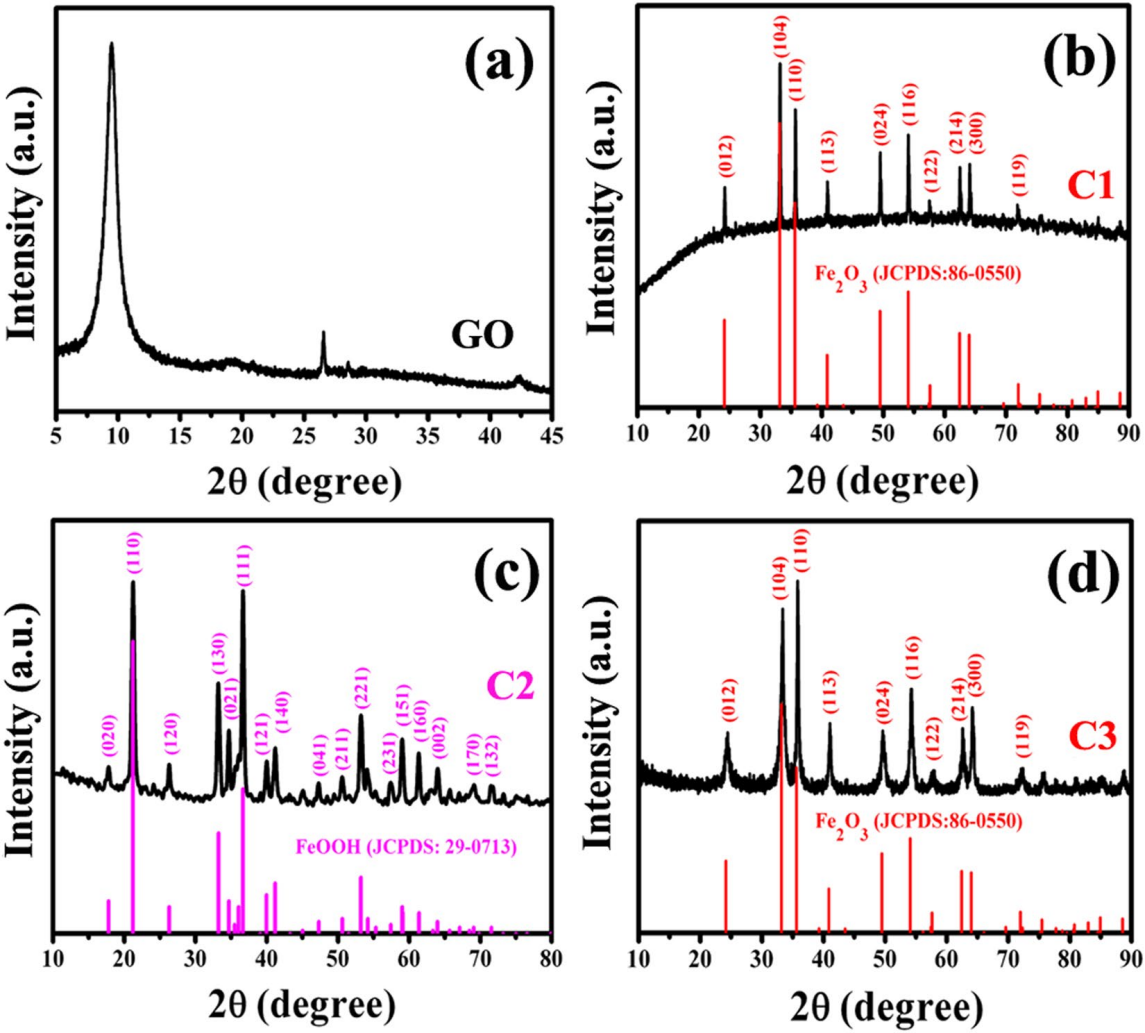
XRD patterns of (**a**) GO, (**b**) C1, (**c**) C2, and (**d**) C3.

Figure 4 presents the XRD patterns of as-synthesized GO and products. As shown in Fig. 4a, the diffraction peaks at 9.4 and 26.5° can be indexed to GO^39, 40^. The diffraction peaks of the as-prepared C1 (As shown in Fig. 4b) located at ca. 24.2, 33.2, 35.7, 41.0, 49.5, 54.1, 57.6, 62.5, 64.1 and 71.9° can be indexed to (012), (104), (110), (113), (024), (116), (122), (214), (300) and (119) facets of hexagonal phase α-Fe_2_O_3_ (JCPDS: 86–0550). For C2, as labeled in Fig. 4c, all the diffraction peaks can be assigned to orthorhombic phase of α-FeOOH (JCPDS: 29–0713). And all the diffraction peaks (as shown in Fig. 4d) appeared in the range of 20–90° can be indexed to hexagonal phase α-Fe_2_O_3_ (JCPDS: 86–0550). Compare the XRD patterns of C1 and C3, one can find that the strongest diffraction peak of C3 is at ca. 35.6° while the strongest one of C1 is at 33.2°. Combined with the TEM results (as shown in Figs 2 and 3), we think the change of the strongest diffraction position may be ascribed to the preferential growth of Fe_2_O_3_ NRs (C3). Based on the obtained TEM and XRD results, one can find that the obtained C1, C2 and C3 are α-Fe_2_O_3_ NPs-RGO, α-FeOOH NRs-RGO and porous α-Fe_2_O_3_ NRs-RGO nanohybrids, respectively.

**Figure 5:**
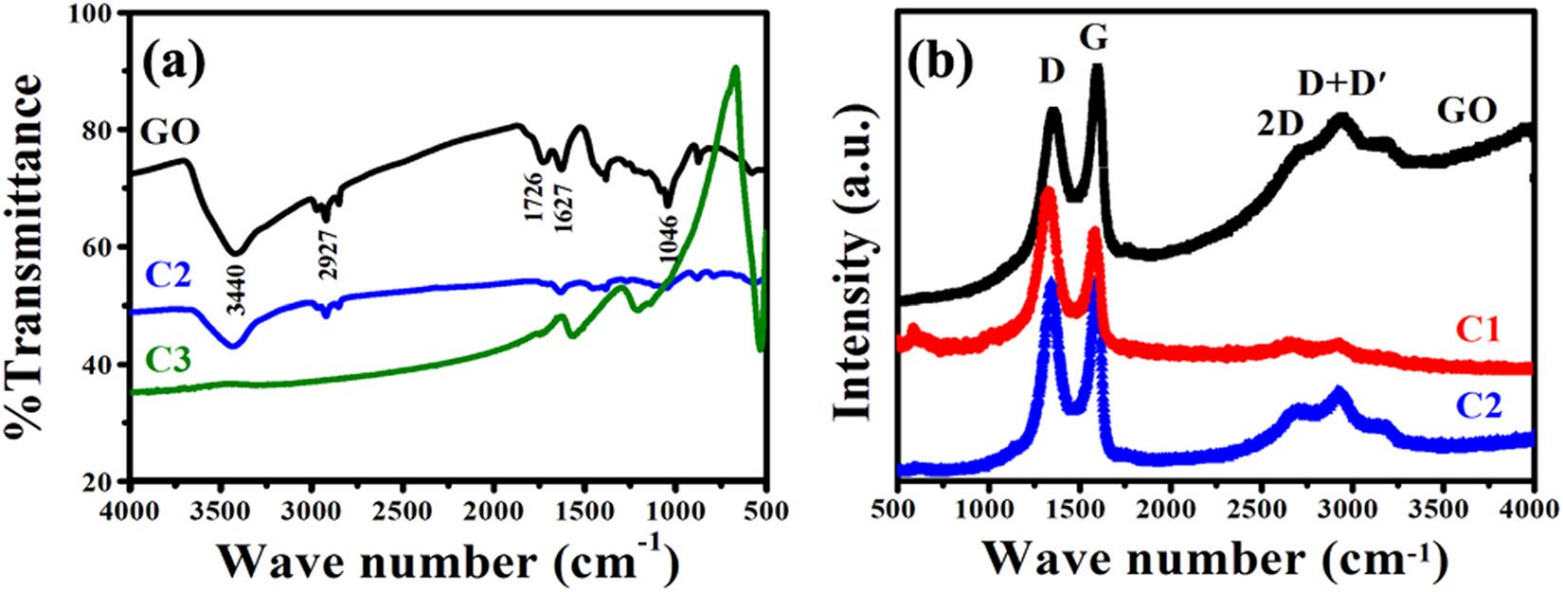
(**a**) FT-IR, and (**b**) Raman spectrum of GO and the as-prepared hybrids.

Figure 5 shows the IR and Raman spectra of the obtained samples. As shown in Fig. 5a, for GO, the peaks at 3440 and 1627 cm^−1^ can be attributed to the stretching vibration of O-H and C = C, respectively. And the other characteristic peaks appeared at 2927, 1726 and 1046 cm^−1^ are due to the stretching vibration of C-H, C = O and epoxy C-O, respectively. Compared to GO, one can find that the oxygen-containing functional groups at 3440, 1726 and 1046 cm^−1^ decrease in the FTIR spectra of the obtained C2 and these peaks almost vanish in the FTIR spectra of the obtained C3, which indicates that the obtained GO is reduced during the hydrothermal process. Figure 5b gives Raman spectra of the as-synthesized GO, C1 and C2, in which two sharp peaks are obviously in common: D band at ca. 1356 cm^−1^ originating from disordered carbon and the G band at ca. 1599 cm^−1^ corresponding to sp^2^ hybridized carbon. Compared with GO, the D band becomes more prominent and an increased D/G intensity ratio of the obtained C1 and C2 can be found, revealing a decrease in the average size of the sp^2^ domains upon further chemical reduction of the GO during thermal synthesis of C1 and C2^41, 42^. We can coarsely evaluate the disorder degree in graphene by the rule: the higher I_D_/I_G_ value, the more defects exist^43^. The increase of I_D_/I_G_ also confirms the reduction of GO to RGO during the reaction process^44^. Furthermore, the 2D peak (2706 cm^−1^) and a combination mode of D + D^´^ at higher wave number (2937 cm^−1^) can also observed clearly over the as-synthesized samples. It is well known that the 2D band in the Raman spectra of graphene is a second-order double-resonance process whose line shape indicates the number of graphene layers in the sample^45–47^.

**Figure 6:**
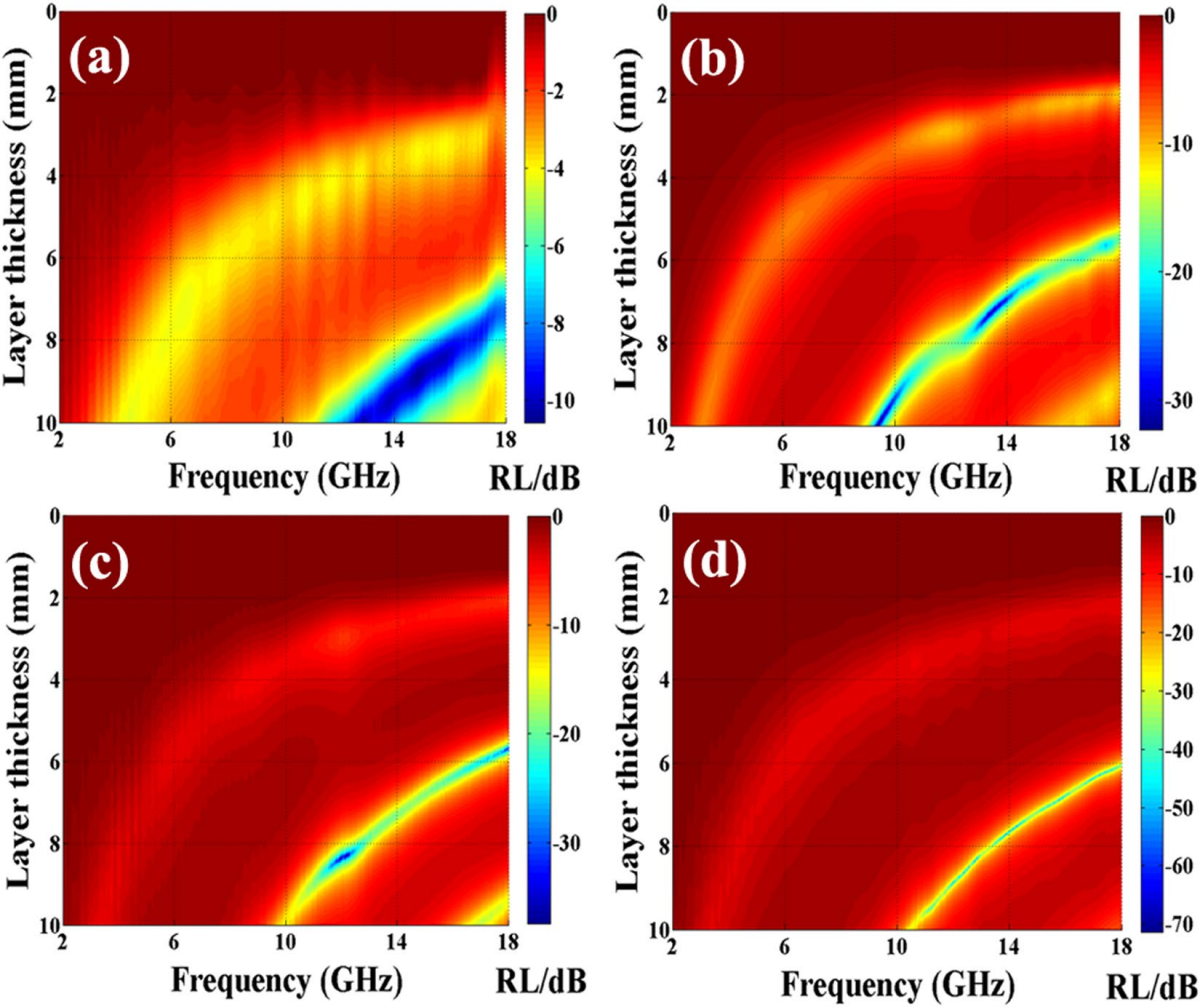
Two-dimensional representation RL values of (**a**) GO, (**b**) C1, (**c**) C2 and (**d**) C3, respectively.

According to the transmission line theory, the values of reflection loss (RL) and attenuation constant (***α***) are calculated by the following equations^48, 49^:

1 $${{\boldsymbol{Z}}}_{{\boldsymbol{in}}}=\sqrt{\frac{{{\boldsymbol{\mu }}}_{{\boldsymbol{r}}}}{{{\boldsymbol{\varepsilon }}}_{{\boldsymbol{r}}}}}\,{\bf{\tanh }}({\boldsymbol{j}}\frac{{\bf{2}}{\boldsymbol{\pi }}\mathrm{fd}\sqrt{{{\boldsymbol{\mu }}}_{{\boldsymbol{r}}}{{\boldsymbol{\varepsilon }}}_{{\boldsymbol{r}}}}}{{\boldsymbol{c}}})$$

2 $${\boldsymbol{RL}}={\bf{20}}\,{\bf{log}}|\frac{{{\boldsymbol{Z}}}_{{\boldsymbol{in}}}{\boldsymbol{-}}1}{{{\boldsymbol{Z}}}_{{\boldsymbol{in}}}{\boldsymbol{+}}1}|$$

3 $${\boldsymbol{\alpha }}=\frac{\sqrt{{\bf{2}}}{\boldsymbol{\pi }}f}{{\boldsymbol{c}}}\sqrt{({\boldsymbol{\mu }}{\boldsymbol{^{\prime\prime} }}{\boldsymbol{\varepsilon }}{\boldsymbol{^{\prime\prime} }}-{\boldsymbol{\mu }}{\boldsymbol{^{\prime} }}{\boldsymbol{\varepsilon }}{\boldsymbol{^{\prime} }})+\sqrt{{({\boldsymbol{\mu }}{\boldsymbol{^{\prime\prime} }}{\boldsymbol{\varepsilon }}{\boldsymbol{^{\prime\prime} }}-{\boldsymbol{\mu }}{\boldsymbol{^{\prime} }}{\boldsymbol{\varepsilon }}{\boldsymbol{^{\prime} }})}^{2}+{({\boldsymbol{\varepsilon }}{\boldsymbol{^{\prime} }}{\boldsymbol{\mu }}{\boldsymbol{^{\prime\prime} }}+{\boldsymbol{\varepsilon }}{\boldsymbol{^{\prime\prime} }}{\boldsymbol{\mu }}{\boldsymbol{^{\prime} }})}^{2}}}$$

where ***f*** is the frequency of EM wave, ***d*** is the thickness of absorber, ***c*** is the velocity of light and ***Z***_***in***_ is the input impedance of absorber. Based on the equations (1) and (2), the RL values of GO, α-FeOOH NRs, the as-synthesized C1, C2 and C3 are obtained. As shown in Fig. 6. It can be seen clearly that: (1) the minimum RL values of the obtained samples move towards the lower frequency region with the increasing thickness; (2) compare GO with α-FeOOH NRs (as shown in Figure S2), the obtained nanohybrids exhibit evidently enhanced microwave absorption performances; (3) the minimum RL values for C1, C2 and C3 are ca. −32.3 dB at 9.4 GHz with the matching thickness of 9.99 mm, −37.4 dB at 12.2 GHz with the matching thickness of 8.29 mm and −71.4 dB at 14.36 GHz with a matching thickness of 7.48 mm, respectively; (4) RL values below −20 dB (99% of EM wave attenuation) for C1, C2 and C3 can be obtained in the frequency range of 12.6–15.1, 11.2–18.0, and 10.3–18.0 GHz, respectively; (5) RL values below −10 dB (90% of EM wave attenuation) for C1, C2 and C3 can be observed in the frequency range of 8.8–18.0, 9.6–18.0 and 9.6–18.0 GHz, respectively. As shown in Table 1, one can find clearly that the as-prepared porous α-Fe_2_O_3_ NRs-RGO hybrids show the superior absorption properties among other similar hybrids.

**Table 1:** EM wave absorption properties of Fe-based nanohybrids reported in recent representative papers.

MAMs (Absorbent content)	Optimum RL (dB)	Frequency range (GHz) (RL < −20 dB)	Frequency range (GHz) (RL < −10 dB)	References
γ-Fe_2_O_3_-MWCNTs^a^ (30 wt%)	−32.7	11.8–13.2	11.3–13.8	50
α-Fe_2_O_3_/CoFe_2_O_4_ (50 wt%)	−60.0	10.0–15.4	8.0–18.0	51
Fe_3_O_4_/G (40 wt%)	−40.36	5.0–13.0	4.2–18.0	52
γ-Fe_2_O_3_/G (20 wt%)	−64.1	6.2–18.0	5.0–18.0	53
α-Fe_2_O_3_/RGO (8 wt%)	−33.5	6.6–13.8	10.8–17.2	54
α-Fe_2_O_3_/G (30 wt%)	−38.0	4.3–14.0	3.8–18.0	55
α-Fe_2_O_3_/G (8 wt%)	−10.0	6.5–15	10.8–17.2	56
α-Fe_2_O_3_/RGO (50 wt%)	−13.6	—	3.2–4.2	36
Fe_3_O_4_/RGO (50 wt%)	−17.1	—	5.5–9.0	36
C1 (30 wt%)	−32.3	12.6–15.1	8.8–18.0	this work
C2 (30 wt%)	−37.4	11.2–18.0	9.6–18.0	this work
C3 (30 wt%)	−71.4	10.3–18.0	9.6–18.0	this work

^a^γ-Fe_2_O_3_-multiwalled carbon nanotubes.

## Discussion

**Figure 7:**
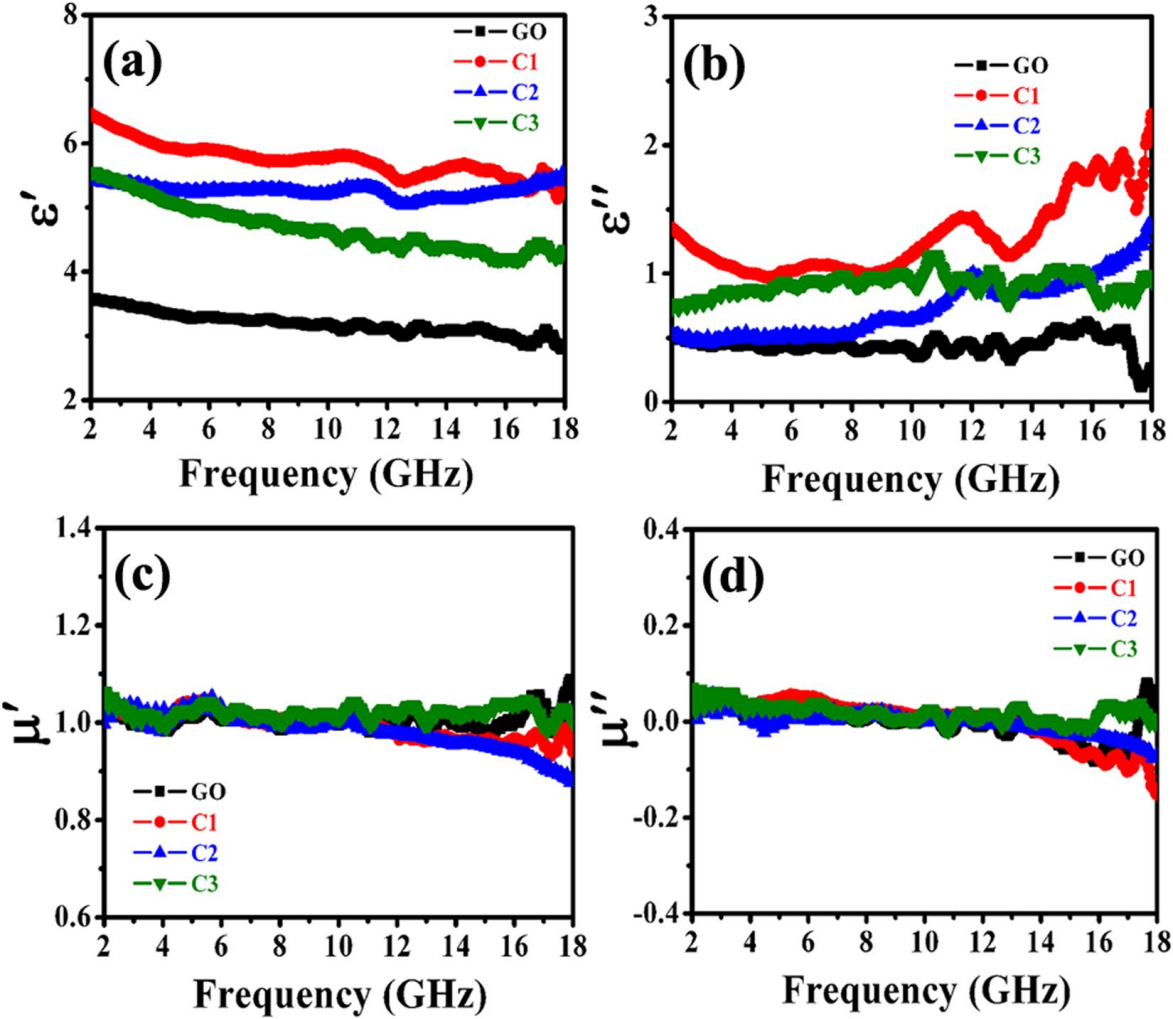
EM characteristics of the obtained hybrids: (**a**,**b**) real and imaginary parts of permittivity, and (**c**,**d**) real and imaginary parts of permeability.

In order to analyze the difference in obtained RL results, the EM parameters, dielectric and magnetic loss abilities, attenuation constant and EM impedance matching are presented. Figure 7 gives the complex permittivity and complex permeability of GO and the as-prepared hybrids in the 2.0–18 GHz frequency range. As shown in Fig. 7a, besides some fluctuations, the ${\boldsymbol{\varepsilon }}{\boldsymbol{^{\prime} }}$ values of the as-synthesized samples are found to decrease with the frequency in the tested region. On the basis of the Debye theory, ${\boldsymbol{\varepsilon }}{\boldsymbol{^{\prime} }}$ can be described as^57^:

4 $${\boldsymbol{\varepsilon }}{\boldsymbol{^{\prime} }}={{\boldsymbol{\varepsilon }}}_{\infty }+\frac{{{\boldsymbol{\varepsilon }}}_{{\boldsymbol{s}}}-{{\boldsymbol{\varepsilon }}}_{\infty }}{{\bf{1}}+{{\boldsymbol{\omega }}}^{{\bf{2}}}{{\boldsymbol{\tau }}}^{{\bf{2}}}}$$

where ${\varepsilon }_{s}$ is the static permittivity, ${\varepsilon }_{\infty }$ is the relative dielectric permittivity at the high frequency limit, ***ω*** is angular frequency, ***τ*** is polarization relaxation time. According to the equation (4), one can find that the decreases of $\varepsilon ^{\prime}$ are mainly attributed to the increase of ***ω***. As reported previously^57, 58^, the phenomenon can be considered as the polarization relaxation in the lower frequency range. It can be seen that the ${\boldsymbol{\varepsilon }}{\boldsymbol{^{\prime} }}$ values of the obtained samples are as follows: α-FeOOH (as shown in Figure S3a) < GO < C3 < C2 < C1. Compared to the previous results of the G-based hybrids, the as-prepared Fe based-RGO nanohybrids exhibit a relatively low ${\boldsymbol{\varepsilon }}{\boldsymbol{^{\prime} }}$ values, which may lead to high impedance matching behavior and good microwave absorption^54, 55, 59^. Although the obtained α-FeOOH and GO exhibit much lower values of ${\boldsymbol{\varepsilon }}{\boldsymbol{^{\prime} }}$, according to the transmission line theory and previous results^48, 60^, single material such as FeOOH, Fe_2_O_3_ or GO cannot exhibit excellent microwave absorption performance. As for the imaginary part of the permittivity (as shown in Figs 7b and S3b), although it has some fluctuations, all in all, it can be seen that the $\varepsilon ^{\prime\prime}$ values of the obtained samples are as follows: α-FeOOH < GO < C2 < C3 < C1. It is well known that the larger value of $\varepsilon ^{\prime\prime}$ indicates an increased dielectric loss^61^. Unlike the dielectric behavior, the permeability properties of the obtained samples are shown in Fig. 7c and d. Overall, there are no significant changes of $\mu ^{\prime}$ and $\mu ^{\prime\prime}$ among the obtained samples, which should be related to their nonmagnetic properties at RT^62^. And the result indicates a small difference of magnetic loss among the obtained nanohybrids. Moreover, as shown in Fig. 7d, we can notice that the $\mu ^{\prime\prime}$ values are negative in part of the frequency range, which may be ascribed to the magnetic energy being radiated out, noise, and/or the permeability-to-permittivity transform of EM wave in nanohybrids^63–67^.

**Figure 8:**
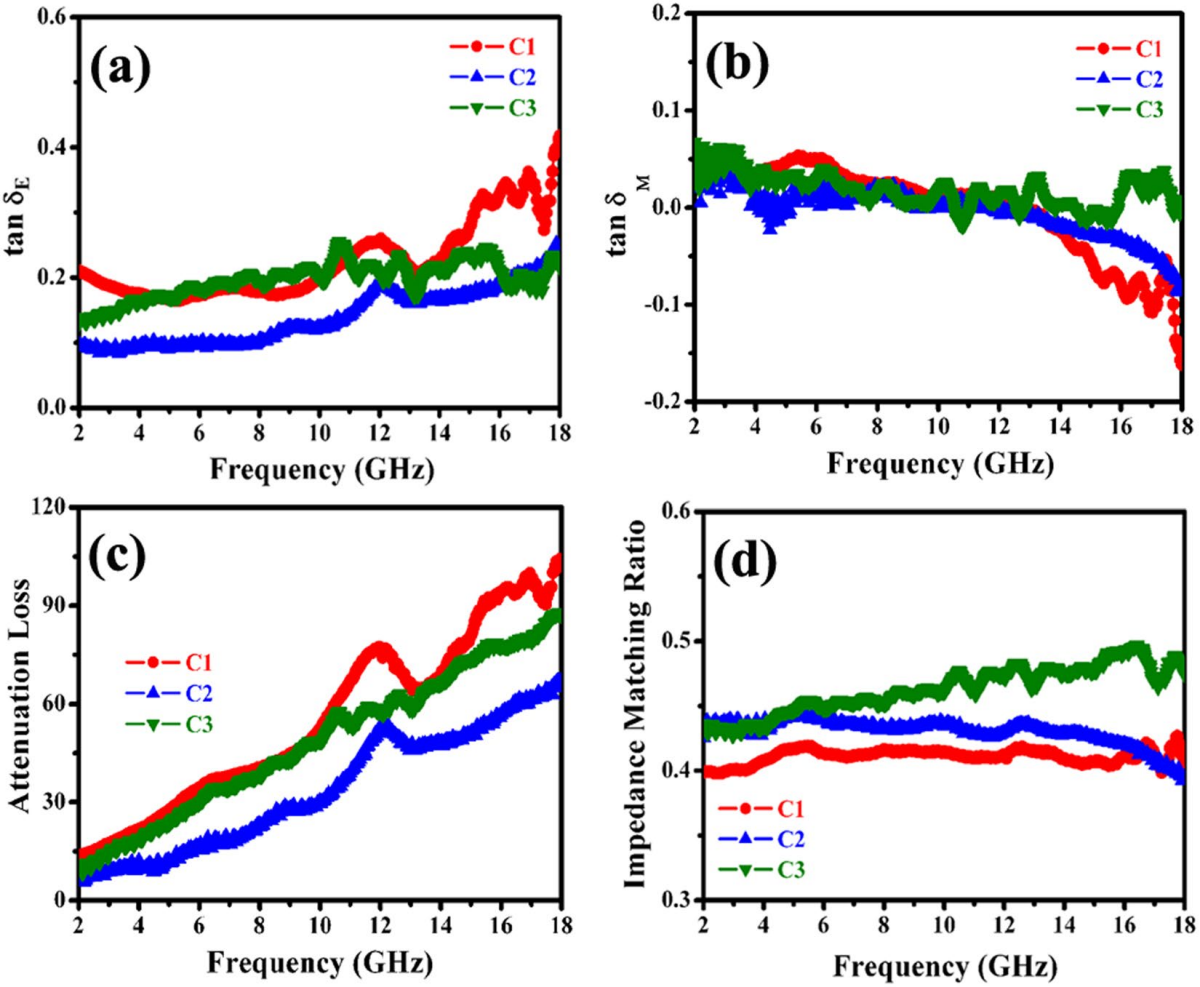
(**a**,**b**) Loss tangent, (**c**) attenuation loss, and (**d**) impedance matching ratios of the as-prepared hybrids.

Figure 8 presents the dielectric and magnetic loss properties, attenuation constant and impedance matching of the obtained nanohybrids. As shown in Fig. 8a and b, one can find that all the obtained hybrids exhibit much larger values of $\tan \,{\delta }_{{\rm{E}}}$ than those of $\tan \,{\delta }_{m}$, which implies that the EM attenuation is mainly due to dielectric loss. And the dielectric loss performance of the hybrids presents the following tendency: C1 > C3 > C2. Moreover, the obtained hybrids display excellent mutual compensation between dielectric loss and magnetic loss, and this effective compensation is very beneficial to enhance their microwave absorption capabilities^68^. According to equation (3), the ***α*** values of hybrids are obtained and shown in Fig. 8c. It can be seen that the as-prepared C1 exhibits the highest ***α*** value while the ***α*** value of C2 is the lowest. In addition, compared to the previously reported MnO_2_@Fe-G^37^, the ***α*** value of the obtained ternary nanohybrids is much higher, and the high value of ***α*** is conducive to improve EM wave absorption capability^49^. Based on the measured complex permittivity and permeability, the impedance matching ratios of the as-prepared hybrids are obtained and displayed in Fig. 8d. As a whole, one can find that the impedance matching ratio of C3 is much higher than those of C1 and C2. It is well known that the excellent impedance matching ratio is favorable to absorb EM wave. Based on the aforementioned results, one can find that the enhanced microwave absorption capabilities of porous α-Fe_2_O_3_ NRs-RGO hybrid can be attributed to the good dielectric loss ability, high attenuation constant and excellent impedance matching ratio.

Recently, two models have been proposed to interpret the excellent EM wave absorption properties of hybrids^30, 49^. The first model is zero reflection, according to the EM wave theory, the relationship ${{\boldsymbol{\mu }}}_{{\boldsymbol{r}}}={{\boldsymbol{\varepsilon }}}_{{\boldsymbol{r}}}$ should be satisfied. However, as shown in Fig. 7, the obtained samples exhibit much higher values of permittivity than their permeability. Therefore, the model cannot be used to explain the obtained results. The other one is geometrical effect, which occurs when the incident and reflected waves in the material are out of phase 180° at the particular thickness. This effect is strongly dependent on the ${\bf{1}}/{\bf{4}}$ wavelength equation^69^

5 $${{\boldsymbol{d}}}_{{\boldsymbol{m}}}={\boldsymbol{nc}}/{\bf{4}}{{\boldsymbol{f}}}_{{\boldsymbol{m}}}\sqrt{|{{\boldsymbol{\mu }}}_{{\boldsymbol{r}}}||{{\boldsymbol{\varepsilon }}}_{{\boldsymbol{r}}}|}\quad \quad ({\boldsymbol{n}}={\bf{1}},{\bf{3}},{\bf{5}}\cdots )$$

**Figure 9:**
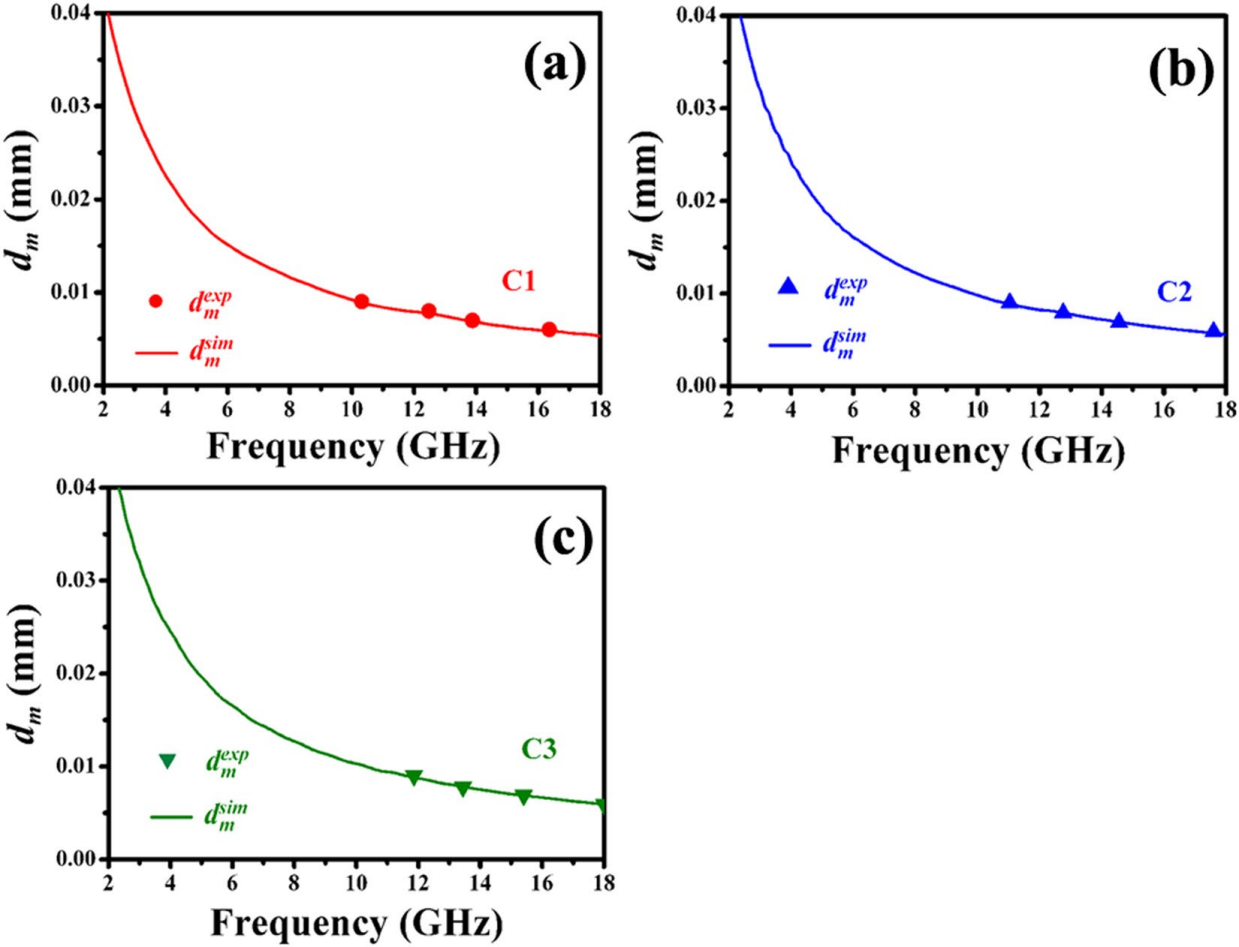
Comparison of the simulated matching thickness (${d}_{m}^{sim}$) under ***n*** = **3** to the ${d}_{m}^{\exp }$ obtained from RL values shown in Fig. 6b–d.

Here, ***d***_***m***_ and ***f***_***m***_ are the matching thickness and peak frequency, $|{{\boldsymbol{\mu }}}_{{\boldsymbol{r}}}|$ and $|{{\boldsymbol{\varepsilon }}}_{{\boldsymbol{r}}}|$ are the modulus of the measured ${\mu }_{r}$ and ***ε***_***r***_ at ***f***_***m***_, respectively. According to equation (5), the ***d***_***m***_ can be simulated, which is denoted as ${d}_{m}^{sim}$, and the results are shownin Fig. 9. It is clearly found that the obtained ${d}_{m}^{sim}$ are in good agreement with the values of ${d}_{m}^{\exp }$ (directly achieved from the RL curves in Fig. 6b–d). Therefore, the excellent microwave absorption properties of Fe based-RGO nanohybrids can be explained by the quarter-wavelength matching model.

**Figure 10:**
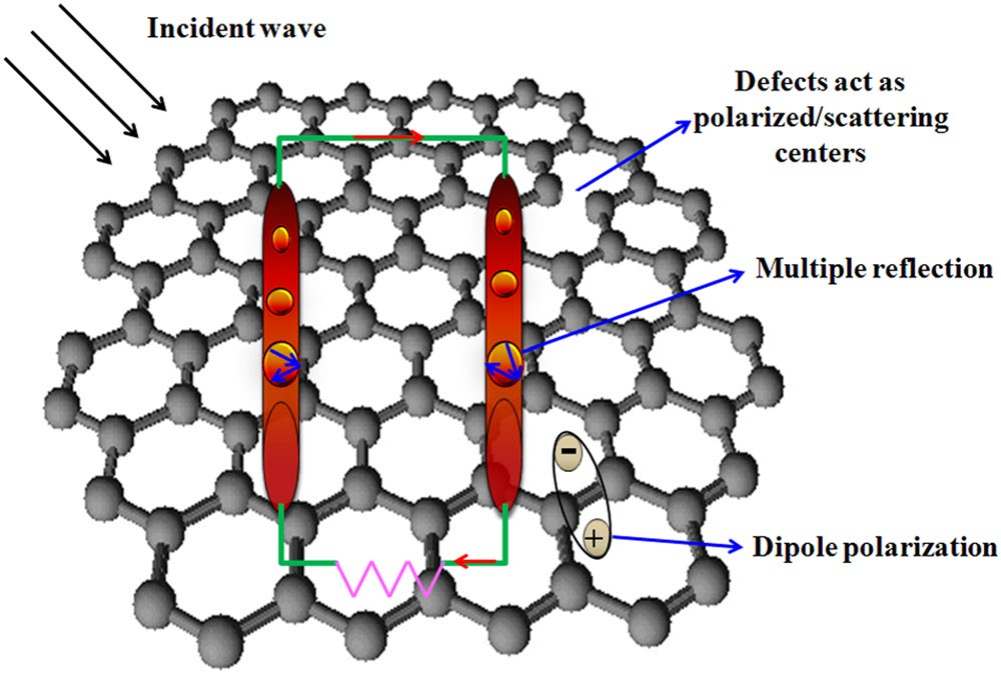
Schematic diagram for possible enhanced microwave absorption mechanism of porous α-Fe_2_O_3_ NRs-RGO hybrid.

Based on the aforementioned results and previous models^37, 55^, the enhanced microwave absorption properties of α-FeOOH NRs-RGO and porous α-Fe_2_O_3_ NRs-RGO hybrids should be related to dielectric loss, conduction loss and multiple reflections in the porous structure of α-Fe_2_O_3_. As schematically shown in Fig. 10, according to the antenna mechanism and obtained results^70^, the rod structure of α-FeOOH and α-Fe_2_O_3_ can be seen as an antenna. When EM wave is projected on this structure, EM wave energy will transfer in form of microcurrent. When the generated current transmits along one rod structure to another, the RGO serves as an electrically conductive network, which can effectively attenuate the EM wave energy. Moreover, as shown in Fig. 5, there are residual oxygen functional groups and defects in the RGO which can act as polarized/scattering centers and enhance the absorption of EM energy. As shown in Figs 3, S1 and S4, the larger BET surface area of porous α-Fe_2_O_3_ NRs-RGO, the interface between RGO and α-Fe_2_O_3_ NRs causes the formation of mangy diploes, interfacial polarization and the associated relaxation, improving the possibility of EM to be absorbed. According to the previous result^55^, the porous α-Fe_2_O_3_ NRs offer an additional opportunity for multiple reflections of the incident wave, which can effectively enhance the ability of EM absorption and attenuation.

In summary, by controlling the categories of the initial reactant, different kinds and morphologies of Fe based-RGO nanohybrids (such as α-Fe_2_O_3_ NPs-RGO, α-FeOOH NRs-RGO and porous α-Fe_2_O_3_ NRs-RGO) can be selectively synthesized by hydrothermal method without using any surfactant and toxic reduced agent. The investigations indicate that the as-prepared Fe based-RGO nanohybrids exhibit excellent microwave absorption properties due to the quarter-wavelength matching model. Moreover, the obtained porous α-Fe_2_O_3_ NRs-RGO nanohybrids exhibit an enhanced microwave absorption performance because of their special structure and synergetic effect, which makes the as-prepared hybrids exhibit good dielectric loss ability, high attenuation constant and excellent impedance matching ratio. The obtained results indicate that the geometrical morphology actually has an important influence on their microwave absorption properties, which may be extended to fabricate other types and morphologies of nanohybrids for high performance MAMs.

## Methods

### Synthesis of products

All the used chemical regents were analytically pure and used without further purification. Firstly, GO was prepared according to the modified Hummers method^71, 72^. After that, 0.05 g of the obtained GO was dispersed into 100 mL deionized water and ultrasonicated for 1 h at room temperature (RT) to obtain suspension liquid. 1.39 g of FeSO_4_·7H_2_O was dissolved in 100 mL deionized water to form a transparent solution. Then 0.084 g NaHCO_3_ was added into the aforementioned GO and FeSO_4_ mixed solution, and the as-synthesized solution was transferred into a 250 mL Teflon-lined stainless steel autoclave and heated at 140 °C for 6 h. After being cooling to RT, the product was separated by centrifugation, washed with distilled water and absolute ethanol, and dried at 60 °C. For easy description, the as-synthesized product was denoted as C1. For comparison, with the other experimental conditions unchanged, the aforementioned solutions of GO and FeSO_4_ (without the introduction of NaHCO_3_) were mixed and sealed into a Teflon-lined stainless steel autoclave for hydrothermal reaction at 140 °C for 6 h. After washed with distilled water, absolute ethanol and dried at 60 °C, the sample (C2) could be collected. Finally, the product (C3) was synthesized through heating C2 sample at 300 °C in N_2_ for 2 h.

### Characterization

The samples were examined on an X-ray powder diffractometer (XRD) at RT for phase identification using CuK_α_ radiation (model D/Max-RA, Rigaku). Raman spectroscopic investigation was performed using a Jobin-Yvon Labram HR800 instrument with 514.5 nm Ar^+^ laser excitation. The morphology investigation was examined using a transmission electron microscope (TEM) (model Tecnai-G20, operated at an accelerating voltage of 20 kV). Fourier transform infrared (FT-IR) spectroscopy of samples (in KBr pellets) was recorded using a Nicolet 510 P spectrometer. For microwave measurement, 30 wt% of the as-prepared sample was mixed with paraffin and pressed into coaxial clapper in a dimension of outer diameter of 7.0 mm, inner diameter of 3.0 mm, respectively. The complex permittivity $({{\boldsymbol{\varepsilon }}}_{{\boldsymbol{r}}}={{\boldsymbol{\varepsilon }}{\boldsymbol{^{\prime} }}}_{{\boldsymbol{r}}}-{\boldsymbol{j}}{{\boldsymbol{\varepsilon }}{\boldsymbol{^{\prime\prime} }}}_{{\boldsymbol{r}}})$ and complex permeability $({{\boldsymbol{\mu }}}_{{\boldsymbol{r}}}={{\boldsymbol{\mu }}{\boldsymbol{^{\prime} }}}_{{\boldsymbol{r}}}-{\boldsymbol{j}}{{\boldsymbol{\mu }}{\boldsymbol{^{\prime\prime} }}}_{{\boldsymbol{r}}})$ of the composites were measured in frequency range of 2–18 GHz over an Agilent E8363B vector network analyzer.

## Electronic supplementary material

Supporting Information
