# Supplementary material for: Preparation of porous Fe2O3 nanorods-reduced graphene oxide nanohybrids and their excellent microwave absorption properties
Source: Sci Rep. 2017 Sep 11;7:11213. doi: 10.1038/s41598-017-11131-1 (PMC5593864; doi:10.1038/s41598-017-11131-1)
Supplement: Supplementary file 1 — Supporting Information [file 41598_2017_11131_MOESM1_ESM.doc]

**Preparation of porous Fe_2_O_3_ nanorods-reduced graphene oxide nanohybrids and their excellent microwave absorption properties**

Qi Hu,^a^ Xiaosi Qi ^a,b^,[[1]](#footnote-2), Hongbo Cai,^a^ Ren Xie,^a^ Liu Long,^a^ Zhongchen Bai,^a^ Yang Jiang,^a^ Shuijie Qin,^a^ Wei Zhong,^b^,[[2]](#footnote-3) Youwei Du^b^

^a^College of Physics, Guizhou University, Guiyang 550025, People’s Republic of China

^b^Collaborative Innovation Center of Advanced Microstructures, Nanjing National Laboratory of Microstructures and Jiangsu Provincial Laboratory for NanoTechnology, Nanjing University, Nanjing 210093, People’s Republic of China

**Experimental section**

**Synthesis of α-FeOOH nanorods.** All the used chemical regents were analytically pure and used without further purification. According to the method reported by Qi et al. ,^1^ In a typical procedure, 1.98 g of FeCl_2_·4H_2_O was added into 50 mL of deionized water under sonication for 30 min to form a homogeneous solution. This solution was transferred into a 100 mL Teflon-lined autoclave and then kept at 90 °C for 12 h. After cooling down naturally, the obtained precipitates were separated by centrifugation and washed with water several times and then dried in vacuum at 60 °C for 12 h to obtain the final products.


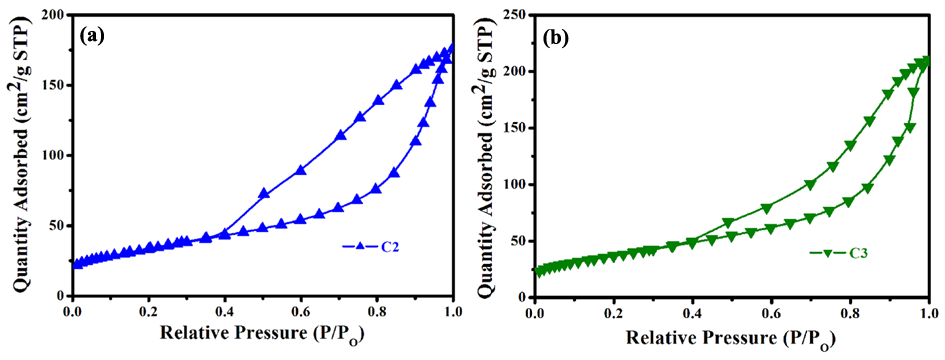


**Figure S1.** N_2_ absorption and desorption isotherms of (a) C2, and (b) C3.


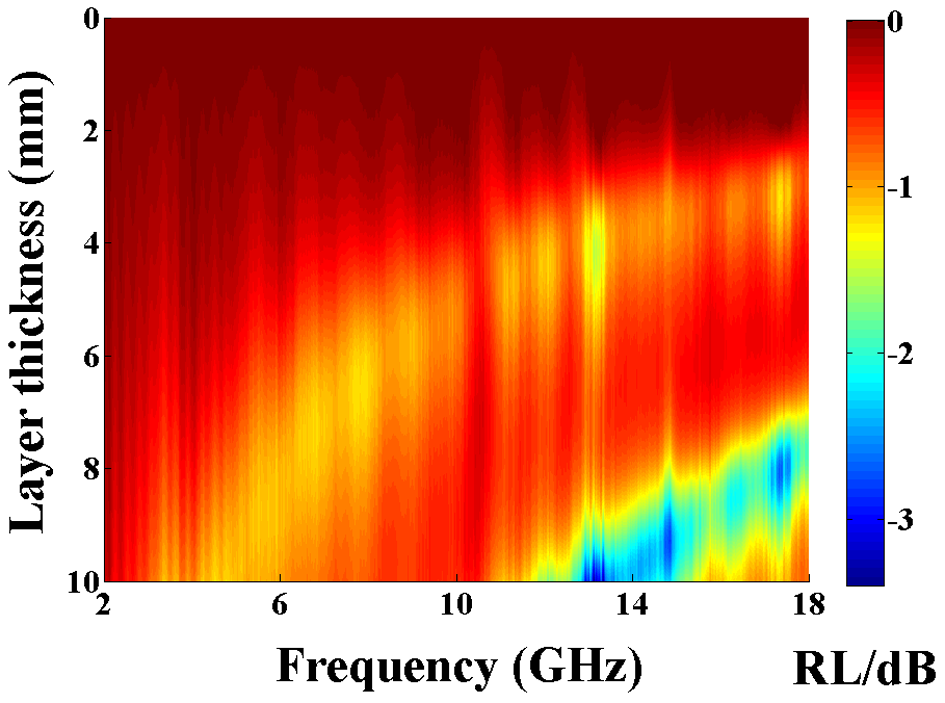


**Figure S2.** Two-dimensional representation RL values of α-FeOOH.


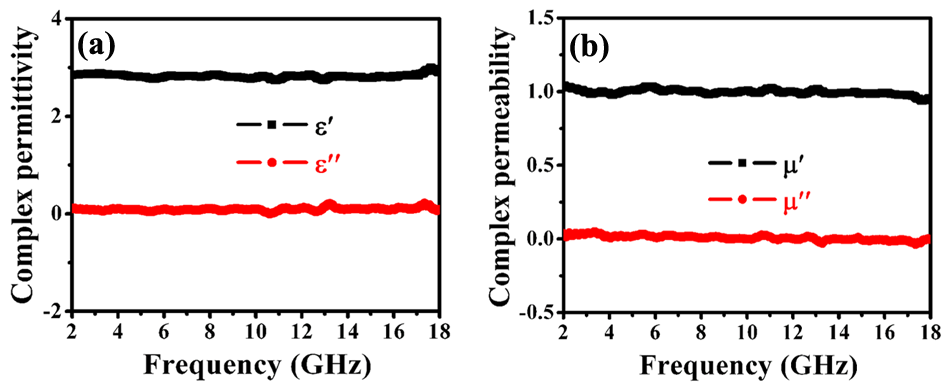


**Figure S3.** (a) Complex permittivity and (b) complex permeability versus frequency of α-FeOOH.


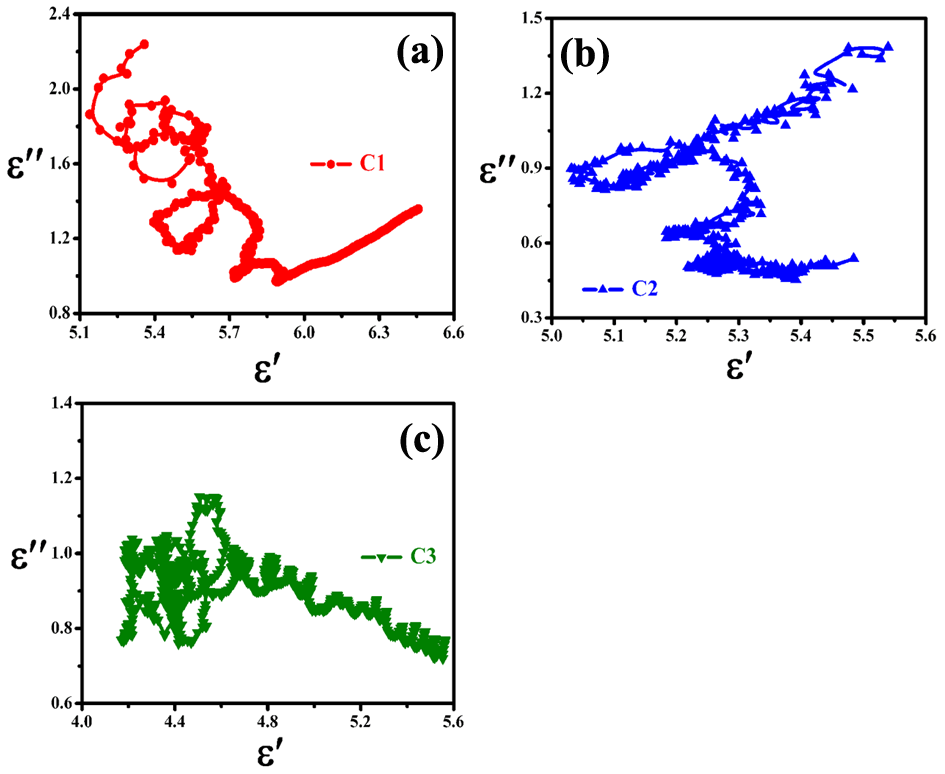


**Figure S4.** Cole-Cole plots of (a) C1, (b) C2 and (c) C3, respectively.

**References**

(1) Qi, H.; Cao, L. Y.; Li, Y. Y.; Huang, J. F. Xu, Z. W. Cheng, Y. Y. Kong, X. G. Yanagisawa, K. High Pseudocapacitance in FeOOH/rGO Composites with Superior Performance for High Rate Anode in Li-Ion Battery. *ACS Appl. Mater. Interfaces,* **2016**, 8, 35253-35263.

1. *****Corresponding author. Phone: +86-25-83621200. Fax: +86-25-83595535

   E-mail: [xsqi@gzu.edu.cn](mailto:xsqi@gzu.edu.cn), [wzhong@nju.edu.cn](mailto:wzhong@nju.edu.cn) [↑](#footnote-ref-2)
2. [↑](#footnote-ref-3)
